# The distribution of school-aged adolescents’ free sugar intake across the day: A cross-sectional study

**DOI:** 10.1038/s41430-026-01714-5

**Published:** 2026-02-27

**Authors:** Marie Murphy, Tessa Hewitt, Abigail Stewart, Breanna Morrison, Rhona Duff, Alice Sitch, Kiya Hurley, Tania Griffin, Ashley Adamson, Suzanne Spence, Maisie Rowland, Peymane Adab, Miranda Pallan

**Affiliations:** 1 Department of Applied Health Sciences, School of Health Sciences, College of Medicine and Health, University of Birmingham, Birmingham, B15 2TT UK; 2 National Institute for Health and Care Research (NIHR) Birmingham Biomedical Research Centre, Birmingham, UK; 3 Department for Health, University of Bath, 1 West, Claverton Down, Bath, BA2 7AY UK; 4 Population Health Sciences Institute, Human Nutrition and Exercise Research Centre, Faculty of Medical Sciences, Newcastle University, Newcastle upon Tyne, NE2 4HH UK

**Keywords:** Nutrition, Epidemiology

## Abstract

**Objective:**

We aimed to explore how free sugar intake varies across the day in secondary school pupils.

**Methods:**

Pupils aged 11-15 years were recruited between December 2019-April 2022 from English secondary schools. Pupils completed a sociodemographic questionnaire and recorded all foods and drinks consumed in the previous day. We undertook mixed-effects regression modelling to explore patterns of sugar consumption across different eating occasions, and in and out of school, accounting for school clustering and adjusting for energy intake and participant sociodemographic variables. 2575 pupils were invited to participate, with 2273 participants ultimately included in the study.

**Results:**

After adjustment for mealtime energy intake, compared to breakfast, free sugar intake was lower at lunch (mean difference: -7.86 g; 95% CI = -8.87 g, -6.84 g; *p* = <0.001) and dinner (mean difference: -11.82 g; 95% CI -12.85 g, -10.80 g; *p* = <0.001). Free sugar intake from snacks was higher than breakfast (mean difference: 7.70 g; 95% CI 6.68 g, 8.72 g; *p* = <0.001). Snacks on average comprised 28.5% of total energy intake and 43.1% of free sugar intake for the day. Free sugar intake outside of school was higher than intake during school time (mean difference: 3.18 g; 95% CI = 1.67 g, 4.69 g; *p* = <0.001).

**Conclusion:**

Breakfast and snacks made the largest contribution to free sugar intake among adolescents in the study after accounting for variation in energy intake across mealtimes. Free sugar intake was higher outside than inside school. Efforts to reduce free sugar intake in adolescents should focus on breakfast and snack food and drink items, and high sugar items available outside of school.

## Introduction

Excessive free sugar intake is closely associated with overweight and obesity, as well as type 2 diabetes, dental caries and various other health issues [1–3]. UK adolescents obtain 12.3% of their total energy intake from free sugars, more than double the recommended amount of 5% [4]. Free sugars are defined in the UK as ‘all added sugars in any form; all sugars naturally present in fruit and vegetable juices, purées and pastes and similar products in which the structure has been broken down; all sugars in drinks (except for dairy-based drinks); and lactose and galactose added as ingredients’ [5]. This definition excludes sugars naturally present in milk/dairy, fresh (and most processed) fruit and vegetables, and cereal grains, nuts and seeds. It therefore provides an estimate of sugars in the diet that are more detrimental to health as they are not bound within cell structures of foods.

Adolescence is an important life stage in which patterns of eating behaviours are formed, which can have profound consequences on an individual’s health throughout their lives [6]. By the age of 11, 41% of UK children are living with either overweight or obesity. This rises throughout adolescence to 64% in adulthood. In addition, almost half of UK adolescents aged 15 years have experienced dental caries [7]. Obesity and dental caries both show a socioeconomic gradient, with higher prevalence associated with higher deprivation [8, 9].

Various policy initiatives have been implemented in the UK to reduce consumption of free sugars, such as the soft drinks industry levy [10] and the national standards for school food [11]. However, the development of policies responding specifically to the needs of adolescents is hampered by the paucity of research on dietary patterns during adolescence [6].

School-aged adolescents consume up to one third of their daily energy intake in school during term-time, so dietary intakes during the school day make an important contribution to overall nutritional status in this age group. School food provision interventions have been shown to have some influence on dietary intake [12, 13], but the evidence for their impact upon nutritional intake within secondary schools is lacking [14]. Understanding patterns of free sugar intake throughout the day, and consumption inside and outside of school, is needed to develop evidence-based interventions to more effectively reduce free sugar intake and improve nutrition in adolescents. In this study we aimed to explore at which eating occasions adolescents attending secondary schools consume the most free sugar, and whether the highest consumption of free sugar is in or outside of school time. We also explored patterns of consumption of sugar sweetened beverages and confectionery.

## Subjects and methods

### Study design and population

We conducted a cross-sectional study using data collected as part of the ‘Food provision, Culture and Environment in Secondary Schools’ (FUEL) study [14]. Secondary schools (academies and free schools) located within the Midlands, England were included in the study. Academies and free schools operate outside of local (but not national) government control and make up more than 80% of secondary schools in England. The Midlands area includes urban and rural areas, has relatively high ethnic diversity [15] and contains areas with high deprivation [16]. We undertook stratified random sampling, based on propensity scores (further details in the FUEL study protocol [14]) and recruited 36 schools. From each school, one class each from Years 7 (age 11–12 years), 9 (age 13–14 years), and 10 (age 14–15 years) was selected to take part. We selected class groupings that were representative of the year group, avoiding classes streamed according to ability or non-core subjects. Informed parental opt-out consent and written assent were obtained from participating pupils. Participants were given a £5 shopping voucher for taking part.

### Data collection and processing

Data were collected from pupils in timetabled school time between November 2019 and April 2022. No data were collected between March 23^rd^ 2020 and June 7^th^ 2021, due to school closures during the COVID-19 pandemic. Dietary intake data including free sugar and energy were collected using Intake24, a web-based self-completion 24-h recall tool based on the multiple pass method [17]. Intake24 has been shown to have similar accuracy to interviewer-led dietary recall in adolescents [18]. Intake24 matches foods and drinks to the National Diet and Nutrition Survey (NDNS) food database, which contains over 2300 foods, and uses nutrient composition data from the UK Nutrient Databank codes [19]. Dietary intake data were collected from pupils using Intake24 for a minimum of one and a maximum of two complete, non-consecutive 24-h periods which included a school day. Using school-provided computers or tablets, pupils entered all foods and drinks consumed at mealtimes and outside of mealtimes (snacks), the location of consumption for each, and whether the food was school provided or brought from home/elsewhere. On the first occasion of pupils completing Intake24, researchers were present to provide a brief explanation of the tool, and to assist if pupils had any questions or issues. Pupils also completed additional online surveys, which included questions relating to their sociodemographic characteristics (date of birth, used to calculate age; gender; ethnicity; home postcode, mapped to Index of Multiple Deprivation (IMD) 2019 scores [20]; and whether they were receiving free school meals (FSM)). Missing ages were imputed using the mean age for the year group (2.6%) and missing IMD scores were imputed using the median IMD rank for pupils in their school (10%). The sample size was estimated based on the primary outcome of the FUEL Study, which aimed to detect a difference of free sugar intake of 4 grams (g) between pupils attending schools either mandated or not mandated to comply with national school food standards. A sample size of 34 schools with 68 pupils per school (*n* = 2317) was estimated to provide 87% power at the 5% significance level [21]. We did not conduct a separate sample size calculation for this study.

Dietary intake and sociodemographic data were matched using unique ID numbers and records were excluded where this was not possible, e.g. missing or incorrect IDs. Intake24 records were excluded if considered implausible, e.g. completed in <2 min, zero daily energy intake or contained non-food/drink entries. An additional step was undertaken to explore outlying values of participant 24-h nutrient intakes by plotting frequency distributions of these variables. For outlying values, the corresponding participant Intake24 records were inspected for potential errors and adjusted if necessary, e.g. implausible portion sizes were edited using information provided in the text by the participant, or where absent by taking the Wrieden average portion values [22].

### Nutritional intake estimation at different eating occasions and in and out of school

Free sugar (grams) and energy intake values (kilojoules), and the number (count) of sugar-sweetened beverages and confectionery items consumed for each participant at different eating occasions and in/out of school were derived from Intake24 records (through matching foods and drinks to the NDNS database). The UK definition of free sugars (set by Public Health England in 2018) was used [5]. Sugar sweetened beverages (SSBs) were defined as those liable for the Soft Drinks Industry Levy (SDIL); containing at least 5 g of sugar per 100 ml in its ready to drink or diluted form [23] within the categories of carbonated drinks, ready-to-drink fruit drinks, cordials & squashes, water, alcohol. Confectionery comprised those items recorded by pupils as sweets and chocolate.

Participants self-selected eating occasions in the Intake24 tool from a pre-determined list which comprised breakfast, lunch, evening meal and four snacking occasions (early, morning, afternoon and late). There was also a free text option for participants to describe the eating occasion in their own words. Free text descriptions were later assigned to one of the pre-determined categories based on the description and timing of the eating occasion. Early and morning snacks and drinks were combined into one eating occasion. Drinks could be entered at any eating occasion.

Food/drinks consumed in school were classified as any items (either school provided or brought from home/elsewhere) recorded as consumed between 9.00 am and 2.00 pm inclusive, and any additional foods or drinks stated as consumed on school premises outside of this period. This definition was used to account for variation in school hours across different schools and on different days of the week. Most participating schools reported having policies in place that prohibited pupils in the relevant year groups from leaving the school grounds at lunchtimes (*n* = 32; no information provided from the remaining 4 schools). All other foods and drinks consumed within the 24-h period were classified as consumed outside of school.

### Statistical analysis

Stata v17 [24] was used for all statistical analyses. Descriptive statistics were used to summarise pupils’ demographic characteristics and nutritional intakes. Where participants had completed two Intake24 surveys across different days, mean values were calculated and used in the descriptive analyses. Initially, energy and free sugar intakes for all participants were summarised for each eating occasion or setting (in/out of school), even when the energy intake for that eating occasion or setting was zero. Energy and free sugar intakes were further summarised after exclusion of records where pupils’ energy intake was zero for an eating occasion or setting.

Free sugar intake was compared across eating occasions and separately across settings (in vs. out of school) using mixed-effects linear regression modelling. For the purposes of the model, all snack occasions across the day were combined into one category. School and individual participant ID were included as random effects in all models to account for clustering of nutritional intake within schools and repeated measures for pupils who completed two separate Intake24 records. Three models were developed for each comparison: unadjusted, adjusted for energy intake at the eating occasion or setting, and fully adjusted (including energy intake, age, gender ethnicity and IMD quintile). Energy intake was included as an adjustment variable to account for the expected variation in energy intake across eating occasions and settings to enable us to explore the relative contribution of free sugar to overall nutritional intake on the different eating occasions/parts of the day.

## Results

### Participant characteristics

**Fig. 1:**
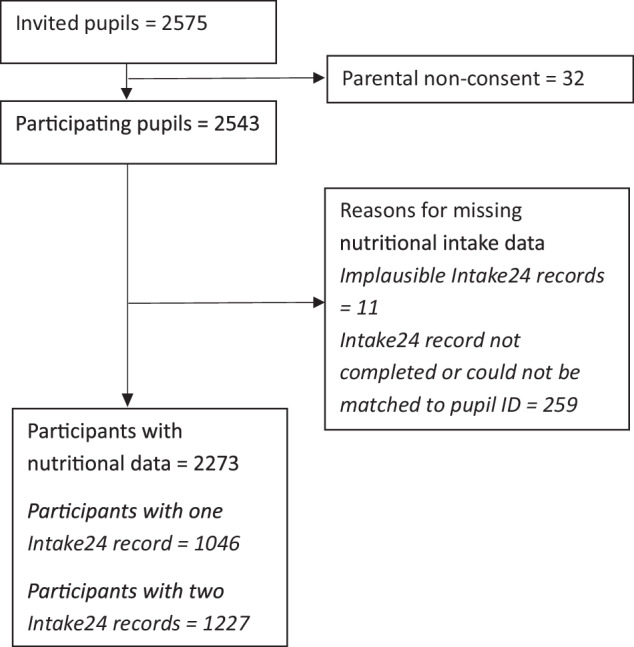
Recruitment of participants.

Across the 36 participating schools, 2543 pupils consented to participate and 2273 (89%) were retained in the current analyses after exclusions were applied (Fig. 1). Of these, 1046 (46.02%) completed two Intake24 records, and the remaining participants completed one Intake24 record.

Participant characteristics are shown in Table 1. There was a slight over-representation of female participants (56%) and participants living in areas of high deprivation (25% in the most deprived quintile). The participant sample was ethnically diverse, with around 70% describing their ethnicity as white, and 12% declared they were receiving FSM.

**Table 1:** Summary of participant demographic data.

	Year 7	Year 9	Year 10	Total
Number (%)	736 (32.38)	796 (35.02)	741 (32.60)	2273 (100.00)
Mean Age (SD)	11.99 (0.36)	13.97 (0.38)	14.97 (0.36)	13.65 (1.28)
Gender (%)				
Male	296 (40.22)	315 (39.57)	317 (42.78)	928 (40.83)
Female	418 (56.79)	456 (57.29)	395 (53.31)	1269 (55.83)
Other^a^/missing	22 (2.99)	25 (3.14)	29 (3.91)	76 (3.34)
Index of Multiple Deprivation Quintile (%)				
1 (most deprived)	185 (25.14)	208 (26.13)	180 (24.29)	573 (25.21)
2	135 (18.34)	131 (16.46)	117 (15.79)	383 (16.85)
3	156 (21.20)	147 (18.47)	158 (21.32)	461 (20.28)
4	146 (19.84)	154 (19.35)	152 (20.51)	452 (19.89)
5 (least deprived)	114 (15.49)	156 (19.60)	134 (18.08)	404 (17.77)
Ethnicity (%)				
White	507 (68.89)	553 (69.47)	516 (69.64)	1576 (69.34)
Asian	116 (15.76)	118 (14.82)	125 (16.87)	359 (15.79)
Black	36 (4.89)	48 (6.03)	39 (5.26)	123 (5.41)
Mixed	37 (5.03)	53 (6.66)	38 (5.13)	128 (5.63)
Other^b^/missing	40 (5.43)	24 (3.02)	23 (3.10)	87 (3.83)
Receiving a Free School Meal (FSM)				
Yes	83 (11.28)	112 (14.07)	74 (9.99)	269 (11.83)
No	430 (58.42)	491 (61.68)	471 (63.56)	1392 (61.24)
Don’t know/ missing	223 (30.30)	193 (24.25)	196 (26.45)	612 (26.92)

^a^Included free-text descriptions of non-binary, genderfluid, agender, gender-queer and questioning

^b^Included Kurdish, English with African backgrounds, Middle Eastern – Persian, English, Arab and British and Asian/Dutch

### Mean energy and free sugar intake by eating occasion and in and outside school

As shown in Table 2, 77% of participants ate breakfast, 92% lunch, 95% evening meal and 89% had one or more snacks during the day. Across the whole sample (including those with zero energy intake at each eating occasion/setting), mean 24-h energy intake was 7399.99 kJ (SD = 3678.74 kJ), and energy intakes were similar at lunch, dinner and combined snacking occasions, but were lower at breakfast. Mean 24-h free sugar intake was 73.36 g (SD = 62.63 g). Mean free sugar intake was approximately equal at breakfast, lunch and dinner, but higher at snacking occasions; with participants consuming almost half of their total daily free sugar intake on these occasions (31.62 g). A similar proportion of pupils consumed food or drink at early/morning, afternoon and late snack times with mean free sugar and energy intake being broadly similar across the snacking occasions.

**Table 2:** Free sugar and energy intake by eating occasion and in and outside school.

	All participants (*n* = 2273)^a^		*N* (%) participants reporting some energy intake	Participants reporting zero energy intake excluded^b^	
	Mean ( ± SD) energy intake (kJ)	Mean ( ± SD) free sugar intake (g)		Mean ( ± SD) energy intake (kJ)	Mean ( ± SD) free sugar intake (g)
24-h intake	7399.99 (3678.74)	73.36 (62.63)			
**Eating occasion**					
Breakfast	1105.83 (1097.13)	13.93 (24.06)	1749 (76.95)	1437.16 (1043.11)	18.11 (26.01)
Lunch	2028.36 (1495.32)	15.10 (19.09)	2088 (91.86)	2208.06 (1427.29)	16.44 (19.36)
Evening meal	2158.28 (1446.95)	12.71 (16.59)	2170 (95.47)	2260.70 (1400.51)	13.31 (16.74)
**Snacks**					
Early/morning	660.28 (1014.70)	8.67 (17.02)	1378 (60.62)	1089.14 (1109.68)	14.30 (19.93)
Afternoon	816.68 (1225.70)	11.82 (21.74)	1435 (63.13)	1293.61 (1327.75)	18.73 (24.89)
Late	630.57 (1059.31)	11.13 (22.43)	1240 (54.55)	1155.83 (1204.20)	20.39 (27.08)
Combined	2107.52 (2265.51)	31.62 (40.53)	2015 (88.65)	2377.39 (2268.94)	35.67 (41.34)
**Setting**					
Intake in school	2807.42 (2082.50)	25.32 (30.67)	2148 (94.50)	2970.81 (2025.77)	26.79 (30.92)
Intake outside of school	4592.57 (2785.62)	48.04 (48.67)	2253 (99.12)	4633.32 (2763.99)	48.47 (48.67)

^a^All participants included in the summary statistics, even if reporting no energy intake at the eating occasion or setting

^b^Participants with zero energy intake at the eating occasion or setting have been excluded in these summary statistics

When participants with zero energy intake were excluded at each eating occasion or setting, free sugar intakes were higher at breakfast than at lunch and dinner, although still less than combined snacking occasions. The distribution of energy intake across eating occasions remained broadly similar. Free sugar intake was higher outside than inside school with mean intakes of 48.47 g (SD = 48.67 g) and 26.79 g (SD = 30.92 g) respectively.

### Intake of sugar sweetened beverages and confectionery

One third of participants (n = 765; 33.66%) consumed at least one SSB item over the 24-h reporting period. A mean of 0.41 (SD = 0.72) SSBs were consumed per participant, equating to 10.69 g (SD = 21.44 g) of free sugar (14% of mean total free sugar intake). A mean of 0.14 (SD = 0.38) SSBs were consumed in school and 0.26 (SD = 0.54) outside school.

Nearly 40% of participants (*n* = 901; 39.64%) consumed at least one confectionery item over the 24-h reporting period. A mean of 0.43 (SD = 0.64) confectionery items were consumed per participant, equating to 12.31 g (SD = 26.80 g) of free sugar. A mean of 0.22 (SD = 0.44) confectionery items were consumed in school and 0.21 (SD = 0.44) outside school.

### Comparison of free sugar intake across different eating occasions and in and out of school using mixed-effects models

The models comparing free sugar intake across eating occasions and the models comparing free sugar intake in and out of school are presented in Table 3. In the model unadjusted for energy intake (model 1), mean free sugar intake was substantially higher from combined snacking occasions than at breakfast (17.39 g; 95% CI: 16.14 g, 18.64 g; *p* = <0.001). Following adjustment for energy intake (model 2), mean free sugar intake from combined snacks remained higher than at breakfast (7.70 g; 95% CI: 6.68 g, 8.72 g; *p* = <0.001) but was 7.86 g lower (95% CI: -8.87 g, -6.84 g; *p* = <0.001) at lunch and 11.82 g lower at dinner (95% CI: -12.85 g, -10.80 g, *p* = <0.001), than at breakfast. Similar differences in free sugar intake were found after further adjustment for sociodemographic characteristics (model 3). Free sugar intake was higher out of school in all models, compared with in school, however the difference was smaller (but remained significant) in the adjusted models (Model 3: 3.18 g; 95% CI: 1.67 g, 4.69 g; *p* < 0.001). Full models are provided in the Supplementary Information.

**Table 3:** Mixed-effects linear regression models to explore the association between free sugar intake and different eating occasions and settings.

	Model 1^a^		Model 2^b^		Model 3^c^	
	Mean difference in free sugar intake (g) (95% CI)	*p* value	Mean difference in free sugar intake (g) (95% CI)	*p* value	Mean difference in free sugar intake (g) (95% CI)	*p* value
**Eating occasion**						
Breakfast	Reference		Reference		Reference	
Lunch	1.30 (0.05, 2.54)	0.04	-7.86 (-8.87, -6.84)	<0.001	-7.86 (-8.88, -6.84)	<0.001
Evening Meal	-1.05 (-2.30, 0.20)	0.10	-11.82 (-12.85, -10.80)	<0.001	-11.83 (-12.85, -10.81)	<0.001
Snacks	17.39 (16.14, 18.64)	<0.001	7.70 (6.68, 8.72)	<0.001	7.69 (6.67, 8.71)	<0.001
**Setting**						
Intake in school	Reference		Reference		Reference	
Intake outside school	22.23 (20.44, 24.02)	<0.001	3.20 (1.69, 4.72)	<0.001	3.18 (1.67, 4.69)	<0.001

^a^School and participant ID are included as random effects, no fixed effects variables

^b^School and participant ID are included as random effects, energy intake for the eating occasion or setting is included as a fixed effect variable

^c^School and participant ID are included as random effects, energy intake for the eating occasion or setting, age, gender ethnicity and IMD quintile included as fixed effects variables

## Discussion

Participants consumed a mean of 73.36 g of free sugar, which substantially exceeds the UK dietary recommendation of a maximum intake of 30 g per day in this age group [25]. This finding reinforces existing research which suggests that adolescents consume substantially above the recommended amount of free sugar and highlights the importance of developing effective interventions which reduce free sugar intake in this population group [26–28].

Adjusting for energy intake, free sugar intake was highest during snacking occasions, suggesting that foods and drinks consumed in between main meals are likely to be higher in free sugar than main meals. An analysis of the UK National Diet and Nutrition Survey (NDNS) data from 2008 to 2016 found that the highest adolescent consumers of sugar were those with the highest intakes of sweetened drinks, fruit juice, cakes, biscuits, sugar and sweet spreads, and chocolate and sugar confectionery [28], which are foods and drinks that are likely to contribute to intake at snack times. Of the main meals, we found that adolescents consumed more free sugar at breakfast than at lunch and dinner after adjustment for energy intake, indicating that foods and drinks high in free sugar may also be a key feature of breakfast. This is consistent with the findings of another analysis using NDNS data from 2014 to 2016, which reported cereals and cereal products (including breakfast cereals) to be a major contributor to free sugar in adolescent diets, as well as sugars, preserves and confectionery, and soft drinks [29]. Breakfast cereals are a popular choice of breakfast food for children and adolescents, but many contain high levels of free sugar [30, 31] which may help to explain our findings.

Most pupils reported consuming a snack or drink outside of mealtimes, which represents an opportunity for intervention to either reduce snack consumption overall, or promote healthier, lower sugar snack/drink items. Breakfast consumption should also be a target for intervention in this age group, although unlike snack intake, regular breakfast consumption is important for overall health and development in children and adolescents [32]. In our study, only three quarters of participants reported having breakfast. Interventions targeting breakfast need to both encourage regular consumption of breakfast and promote consumption of foods and drinks that are lower in sugar. One strategy to address the latter is food reformulation. In the UK, the Soft Drinks Industry Levy (SDIL) has had some effect in prompting the industry to reformulate their products. An interrupted time series analysis of changes in soft drink purchasing before and after the introduction of the SDIL demonstrated a 8 g reduction in sugar intake per household per week despite an increased volume of soft drink purchased [10]. A similar approach could be applied breakfast cereal manufacturers, whereby they are incentivised to lower the sugar content of their products [31]. The UK government introduced a voluntary sugar reduction programme for the food industry in 2017, but this resulted in only minimal reductions in the sugar content of targeted foods [33]. Another potential intervention to target breakfast consumption in this age group is universal school breakfast provision. If implemented in combination with school food standards that limit the availability of foods and drinks high in sugar, this strategy could promote regular consumption of healthy breakfast foods in this age group. In England, the government are introducing universal free breakfast clubs across all primary schools (children aged 4–11 years) [34] but this does not currently extend to secondary schools.

Free sugar intake outside of school was marginally higher than inside school, which indicates that foods and drinks consumed outside of the school day may be greater contributors to high free sugar intakes. An Australian cross-sectional study conducted with secondary school students found that snacking behaviours were highest in the after-school period [35], which may explain the higher free sugar intake outside of school that we observed. It may, therefore, be important to address snacking and drinking behaviours outside of the school day to effectively reduce intakes of free sugar in adolescents. To date, most interventions aimed at reducing sugar intakes in adolescents have focused on the school setting [36].

SSB intake was higher outside school, but a similar number of confectionery items were consumed inside and outside of school. The current English school food standards [11] prohibit the sales of SSBs and sugar and chocolate confectionery in school, and the limited availability of these items may contribute to reducing consumption of these foods and drinks in school. However, the school food standards do not apply to food and drink items brought into school by pupils. In addition, our evaluation of secondary school compliance with the national standards (undertaken as part of the FUEL study [21]) indicated variable compliance with confectionery-related standards. These factors may help to explain the similar intakes of confectionery inside and outside of school.

To address adolescents’ sugar intake both in and out of school, a systems approach that encompasses all the environments that adolescents interact with is required [37]. Schools are vital partners in this approach, but need to be supported by other agencies and structures to effect change in adolescents’ dietary intake.

Key strengths of the study were the large regional sample, demographically representative of the English population. Dietary intake was measured by 24-h recall, a suitable method for this age group [22] and in nearly half of the participants, we measured dietary intake on two separate days. Limitations included the accuracy of self-reported dietary intake. Underreporting is a known issue in all self-reported dietary assessment, particularly for some nutrients e.g. energy intake [38]. As a result, the adjustment of our models for energy intake may not have fully accounted for its influence upon sugar free intakes. However, underreporting with Intake24 is similar to that using interviewer-led 24-h recall [18]. The use of pre-defined mealtime labels (breakfast, lunch, evening meal, snack/drink) may not fully reflect adolescent eating patterns, however, this was arguably the most practical approach available. The methods we used for defining dietary intake inside and outside school may have led to some misclassification, as these were based in part on time of consumption during the day. However, the impact of this upon the results is likely to be minimal.

Given the findings of this study, it is recommended that future interventions and policies to reduce free sugar intake among adolescents focus on snacking occasions (including drinks outside of mealtimes) and breakfast. There is also a need to focus future intervention efforts outside of the school setting, particularly given that most interventions targeting this age group to date have focused on schools and school food. A further understanding of the relative influence of home versus other out-of-school contexts upon dietary behaviours would help in developing more effective strategies to reduce free sugar intakes in this age group.

## Supplementary information

Supplementary Information: Full mixed effects models

## Data Availability

Data are available from the corresponding author on reasonable request.
