# Supplementary material for: The distribution of school-aged adolescents’ free sugar intake across the day: A cross-sectional study
Source: Eur J Clin Nutr. 2026 Feb 27;80(4):427–33. doi: 10.1038/s41430-026-01714-5 (PMC13083234; doi:10.1038/s41430-026-01714-5)
Supplement: Supplementary file 1 — Supplementary Information: Full mixed effects models [file 41430_2026_1714_MOESM1_ESM.docx]

Supplementary Information: Full mixed effects models

*Table A1.1: Models to explore the association between eating occasion and free sugar intake (in grams)*

|  | Model 1 (unadjusted) | | Model 2 (adjusted for energy intake in kJ) | | Model 3 (adjusted for energy intake in kJ, age, in years, IMD quintile, gender and ethnicity) | |
| --- | --- | --- | --- | --- | --- | --- |
|  | Regression coefficient (95% CI) | p value | Regression coefficient (95% CI) | p value | Regression coefficient (95% CI) | p value |
| Meal time (reference: Breakfast)  Lunch  Dinner  Snacks | 1.30 (0.05, 2.54)  -1.05 (-2.29, 0.20)  17.39 (16.14, 18.64) | 0.04  0.10  <0.001 | -7.86 (-8.87, -6.84)  -11.82 (-12.85, -10.80)  7.70 (6.68, 8.72) | <0.001  <0.001  <0.001 | -7.86 (-8.88, -6.84)  -11.83 (-12.85, -10.81)  7.69 (6.67, 8.71) | <0.001  <0.001  <0.001 |
| Energy intake (kJ) |  |  | 0.185 (0.179, 0.190) | <0.001 | 0.185 (0.180, 0.190) | <0.001 |
| Age (years) |  |  |  |  | -0.47 (-0.82, -0.12) | 0.01 |
| IMD quintile (reference: 1 – most deprived)  2  3  4  5 (least deprived) |  |  |  |  | -0.03 (-1.51, 1.45)  0.06 (-1.46, 1.58)  -0.19 (-1.77, 1.38)  -0.36 (-1.99, 1.28) | 0.97  0.94  0.81  0.67 |
| Gender (reference: male)  Female  Other / not specified |  |  |  |  | 0.81 (-0.15, 1.78)  -0.64 (-3.18, 1.90) | 0.10  0.62 |
| Ethnicity (reference: white)  Asian  Black  Mixed  Other / not specified |  |  |  |  | -2.68 (-4.02, -1.34)  0.46 (-1.63, 2.56)  -1.59 (-3.56, 0.38)  -0.34 (-2.78, 2.09) | <0.001  0.66  0.11  0.78 |

All models included participant ID and school ID as random effects

*Table A2.2: Models to explore the association between setting (in vs out of school) and free sugar intake (in grams)*

|  | Model 1 (unadjusted) | | Model 2 (adjusted for energy intake in kJ) | | Model 3 (adjusted for energy intake in kJ, age, in years, IMD quintile, gender and ethnicity) | |
| --- | --- | --- | --- | --- | --- | --- |
|  | Regression coefficient (95% CI) | p value | Regression coefficient (95% CI) | p value | Regression coefficient (95% CI) | p value |
| Intake location (reference: intake in school)  Intake out of school | 22.23 (20.44, 24.02) | <0.001 | 3.20 (1.69, 4.72) | <0.001 | 3.18 (1.67, 4.69) | <0.001 |
| Energy intake (kJ) |  |  | 0.184 (0.180, 0.188) | <0.001 | 0.184 (0.180, 0.188) | <0.001 |
| Age (years) |  |  |  |  | -0.94 (-1.64, -0.24) | 0.009 |
| IMD quintile (reference: 1 – most deprived)  2  3  4  5 (least deprived) |  |  |  |  | -0.08 (-3.03, 2.87)  0.18 (-2.85, 3.21)  -0.37 (-3.51, 2.77)  -0.72 (-3.98, 2.54) | 0.957 0.907 0.818 0.666 |
| Gender (reference: male)  Female  Other / not specified |  |  |  |  | 1.87 (-0.56, 3.79)  -0.85 (-5.91, 4.21) | 0.057 0.742 |
| Ethnicity (reference: white)  Asian  Black  Mixed  Other / not specified |  |  |  |  | -5.31 (-7.98, -2.64)  0.81 (-3.35, 4.98)  -3.14 (-7.05, 0.77)  0.81 (-5.65, 4.03) | <0.001  0.702 0.116 0.743 |

All models included participant ID and school ID as random effects
